# Pelvic Inflammatory Disease in the Form of Peritoneal Abscess Complicating Late Pregnancy

**DOI:** 10.1155/2011/851598

**Published:** 2011-07-18

**Authors:** H. Mahesha Navada, B. Poornima Ramachandra Bhat

**Affiliations:** Department of Obstetrics and Gynaecology, Father Muller Medical College, Mangalore, Karnataka 565002, India

## Abstract

Chronic pelvic inflammatory disease (PID) in the form of peritoneal abscess during pregnancy is almost unheard entity. Early and antenatal diagnosis may be difficult or may be revealed only during caesarean section. We report a case of peritoneal abscess secondary to pelvic inflammatory disease incidentally found at repeat caesarean section. It was managed by conservative surgical approach.

## 1. Introduction

Pelvic and peritoneal abscess complicating pregnancy is rare. Although nongynaecological conditions contribute to the aetiologies of this unusual complication during pregnancy, rarely it may also be a complication of chronic pelvic inflammatory disease. As the classical clinical features are altered by the gravid uterus, the antenatal diagnosis of chronic PID is difficult. The delayed diagnosis and management may be detrimental to both mother and foetus. We present a case of peritoneal abscess secondary to chronic PID which was found incidentally at repeat caesarean section.

## 2. Case Report

**Figure 1:**
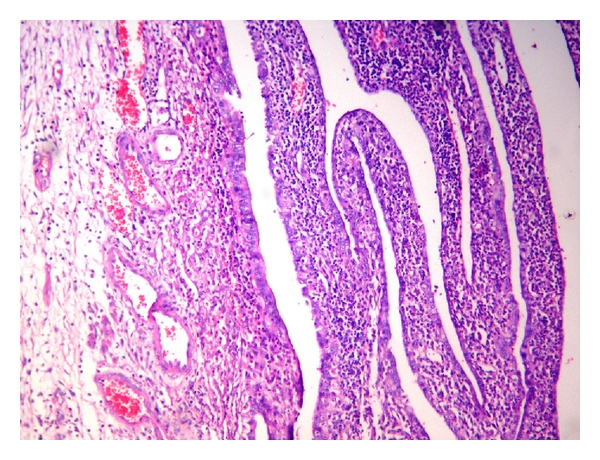
×4 power H&E view thickened mucosal folds of fallopian tube.

**Figure 2:**
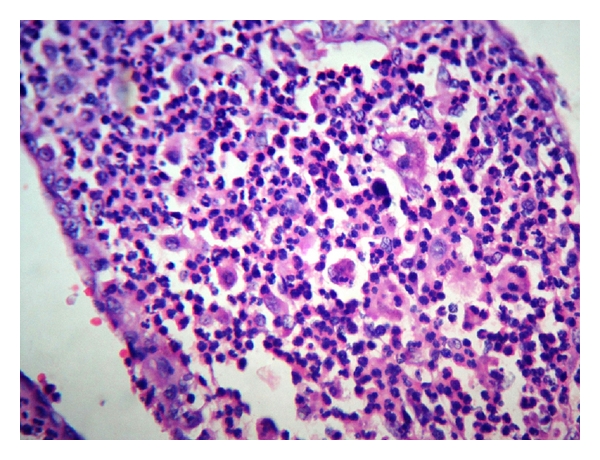
×40 power H&E view inflammatory infiltrate of the fallopian tube.

**Figure 3:**
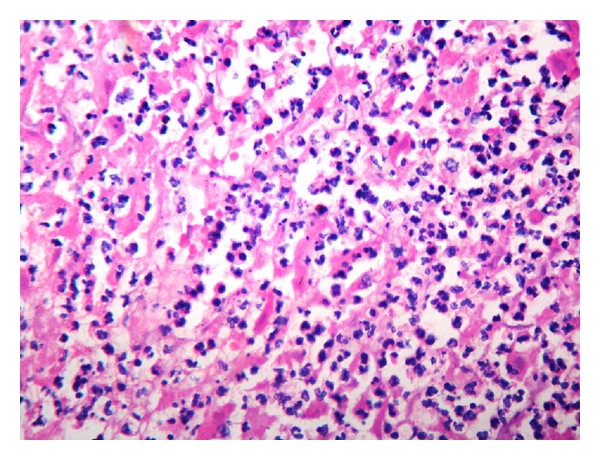
×40 view H&E corpus luteum with neutrophils.

A 36-year-old woman with gravida 2 Para 1 Living 1 with previous caesarean section at 29 weeks four days of gestation was referred with diffuse pain abdomen and giddiness. The pain was diffuse and present for the past 20 days which was partially relieved by symptomatic treatment by local doctors, and she also received antibiotics suspecting urinary tract infection. However she presented then with history of giddiness and one episode of vomiting with no history of diarrhoea and was referred. She was married for 10 years. Her first pregnancy was three years back when she underwent caesarean section for placenta previa. She continued to have episodes of pain abdomen in the postnatal and interpregnancy period for which she received symptomatic treatment by local doctor. She was pale on examination. Her vital signs including temperature were normal. There was no guarding or rigidity except mild tenderness on abdominal examination. The uterus was corresponding to 28-week gravid uterus size and was showing occasional contractions but no scar tenderness. Foetus was in breech presentation having regular heart rate. Her investigations showed mild anaemia with haemoglobin of 8.7 g% and neutrophilic leukocytosis (WBC 20900/c mm). Obstetric ultrasound showed single live intrauterine foetus corresponding to 29- to 30-week gestation. Suspecting chorioamnionitis the tocolysis was not administered. She went into spontaneous preterm labour. She was posted for emergency caesarean in view of previous caesarean with breech presentation under spinal anaesthesia with the coverage of antibiotics. On opening the abdomen, multiple pockets of pus were noted in the peritoneal cavity, and the coils of intestine and omentum were found adherent to the fundus and anterior upper surface of the body of the uterus. Lower segment caesarean section was done, and live male baby weighing 1.37 kg was extracted as breech. Then the general anaesthesia was induced, and the exploration was done by extending the incision. The adhesion between loops of intestine and uterus was released. Loculated pus was drained from subhepatic and subphrenic spaces. No bowel perforation was noted even after thorough exploration. The right salphinx and ovary looked congested and unhealthy covered all over by pus. The left tube and ovary were normal. The right salpingo-oophorectomy and appendicectomy were done. Peritoneal lavage was given, and the abdomen was closed in layers. Patient was discharged on 14th postoperative day in healthy condition. Histopathological examination of fallopian tube showed areas of haemorrhage (Figure 1) and lymphoplasmacytic inflammatory infiltrate (Figure 2), and that of the ovary showed chronic inflammatory infiltrate (Figure 3) suggestive of pelvic inflammatory disease. Section from appendix showed features of only lymphoid hyperplasia. Aerobic organisms were not grown in the culture obtained from the pus from the peritoneal cavity. She had come for follow-up later with postnatal period uneventful. 

## 3. Discussion

Pelvic inflammatory disease in the form of pelvic and peritoneal abscess complicating pregnancy is rare. Blanchard et al. found that acute salpingo oophorities during pregnancy occurs more commonly in first trimester [1]. Sherer DM et al. have reported a recurrent pelvic abscess in pregnancy and in thier review noted that the pelvic infection and pelvic abscess are less common in second and third trimester than in cases diagnosed in the first trimester [2]. On the other hand pelvic infection readily occurs in the puerperium if there is infection of the birth canal during or following parturition. Similar infection can also occur from surgical site infection following caesarean section. Ascending infection is the most important mode of infection in nonpregnant women. However during pregnancy, pelvic infection occurs quite independent of the gravid state or the infection may exist before the pregnancy. The aetiologies may include nongynecological conditions such as ruptured diverticulitis or appendicitisi; tubo-ovarian abscess of unknown origin has also been reported [3]. Friedman and Bobrow have proposed four mechanisms for infection of ovaries during pregnancy [4].

1. Haematogenous spread as in pelvic tuberculosis.
2. Lymphatic spread especially from vagina and cervix.
3. Infection of a previously existing ovarian cyst.
4. Flare-up of old infection.

It is difficult to state the exact mechanism of development of pelvic infection in our case. At laparotomy there was no clear-cut evidence of spread of infection from adjacent organs, but the preexisting salpingitis or previously ruptured tubo-ovarian abscess could not be ruled out. Thus the proposed mechanism in our patient could be flared up of old infection. Intraoperative finding of adhesion between the loops of intestine, the uterus, and the tubo-ovarian tissue indicates the chronic nature of the disease. Hence she might have contracted the pelvic infection before pregnancy following the first caesarean section. Recurrent symptoms of abdominal pain in the postoperative period and inter pregnancy period suggest the chronic nature of the disease. However the courses of antibiotics and the low virulence of the organisms resulted in chronic pelvic infection. The infection might have flared up recently and presented in the third trimester of pregnancy with some acute symptoms like abdominal pain with giddiness and vomiting to suggest peritonitis. Patients with PID during pregnancy may present with wide range of clinical symptoms, and the findings may be altered significantly by the size of the gravid uterus [2]. As there were no classical clinical features of acute peritoneal or pelvic infection, it was not suspected preoperatively and was detected only during caesarean section.

As most of the patients are young, we should attempt conservative surgery if the pathology is limited to one adnexa [3]. Surgical drainage of ovarian abscess and conservative surgical approach under antibiotics are recommended during pregnancy albeit there is no consensus on patient management [5]. Our patient had one-sided adnexal disease. So, unilateral salpingo oophorectomy was done along with peritoneal lavage conserving the other tube and ovary.

In conclusion, PID should be considered in a differential diagnosis of abdominal pain even in pregnancy in spite of its rarity. Prompt and early diagnosis is mandatory for decreasing maternal and perinatal morbidity and mortality.
